# Blood culture-PCR to optimise typhoid fever diagnosis after controlled human infection identifies frequent asymptomatic cases and evidence of primary bacteraemia

**DOI:** 10.1016/j.jinf.2017.01.006

**Published:** 2017-04

**Authors:** Thomas C. Darton, Liqing Zhou, Christoph J. Blohmke, Claire Jones, Claire S. Waddington, Stephen Baker, Andrew J. Pollard

**Affiliations:** a Oxford Vaccine Group, Department of Paediatrics and the NIHR Oxford Biomedical Research Centre, University of Oxford, Oxford, United Kingdom; b The Hospital for Tropical Diseases, Wellcome Trust Major Overseas Programme, Oxford University Clinical Research Unit, Ho Chi Minh City, Viet Nam; c Centre for Tropical Medicine and Global Health, Nuffield Department of Clinical Medicine, Oxford University, Oxford, United Kingdom

**Keywords:** Typhoid fever, Controlled human infection model, Diagnostics, Polymerase chain reaction, Febrile disease, *Salmonella* Typhi

## Abstract

**Background:**

Improved diagnostics for typhoid are needed; a typhoid controlled human infection model may accelerate their development and translation. Here, we evaluated a blood culture-PCR assay for detecting infection after controlled human infection with *S*. Typhi and compared test performance with optimally performed blood cultures.

**Methodology/Principal findings:**

Culture-PCR amplification of blood samples was performed alongside daily blood culture in 41 participants undergoing typhoid challenge. Study endpoints for typhoid diagnosis (TD) were fever and/or bacteraemia. Overall, 24/41 (59%) participants reached TD, of whom 21/24 (86%) had ≥1 positive blood culture (53/674, 7.9% of all cultures) or 18/24 (75%) had ≥1 positive culture-PCR assay result (57/684, 8.3%). A further five non-bacteraemic participants produced culture-PCR amplicons indicating infection; overall sensitivity/specificity of the assay compared to the study endpoints were 70%/65%. We found no significant difference between blood culture and culture-PCR methods in ability to identify cases (12 mismatching pairs, *p* = 0.77, binomial test). Clinical and stool culture metadata demonstrated that additional culture-PCR amplification positive individuals likely represented true cases missed by blood culture, suggesting the overall attack rate may be 30/41 (73%) rather than 24/41 (59%). Several participants had positive culture-PCR results soon after ingesting challenge providing new evidence for occurrence of an early primary bacteraemia.

**Conclusions/Significance:**

Overall the culture-PCR assay performed well, identifying extra typhoid cases compared with routine blood culture alone. Despite limitations to widespread field-use, the benefits of increased diagnostic yield, reduced blood volume and faster turn-around-time, suggest that this assay could enhance laboratory typhoid diagnostics in research applications and high-incidence settings.

## Highlights

• Culture in ox-bile/tryptone soy broth selectively enriches for bile-tolerant *Salmonella* Typhi while lysing human cells.
• PCR sensitivity for detecting typhoid in clinical blood is limited by very low level bacteraemia during clinical illness.
• PCR amplification of *S.* Typhi *fliC*-d in pre-cultured blood can accurately identify typhoid infection in challenge study participants.
• Daily culture-PCR of blood collected from challenge study participants suggests primary bacteraemia occurs 12–36 h after *S.* Typhi ingestion.
• Additional use of culture-PCR demonstrates the true attack rate after typhoid challenge is markedly higher (75%) than previously assumed (60%).

## Graphical abstract

The culture-PCR assay for detection of *fliC-*D DNA in human blood samples.

**Figure:**
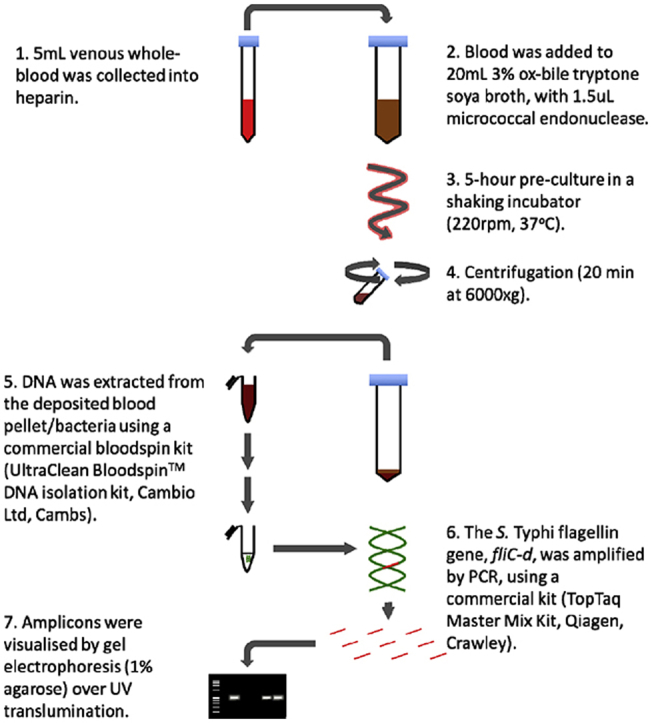

## Introduction

Typhoid fever, a non-specific febrile illness caused by infection with *Salmonella enterica* serovar Typhi (*S.* Typhi), is common in tropical regions.^1^ A key limitation to improving the control of typhoid fever is the lack of reliable diagnostic tests.^2^, ^3^ In addition to confirming infection in individuals, accurate laboratory diagnostics are needed to ascertain true disease burden, to improve understanding of the natural history of infection in humans, and to evaluate vaccine efficacy.^1^, ^2^, ^4^

Diagnostic approaches for typhoid infection are broadly aimed either at directly detecting bacteria or bacterial products or measuring the host response in clinical samples.^2^, ^4^, ^5^ Blood culture remains the diagnostic technique of choice, but only identifies 45–70% of confirmed cases, even with the availability of newer continuous automated culture systems.^5^, ^6^, ^7^ Serological tests including the Widal test are widely available in endemic settings, although in the absence of paired clinical samples or background population serosurveillance data these tests perform poorly with low sensitivity and specificity.^5^, ^8^

Given the poor accuracy of currently available diagnostic tests, attempts have been made to develop PCR-based assays to detect bacterial DNA.^9^, ^10^, ^11^, ^12^, ^13^ Few, if any, of these approaches have been instituted in clinical settings mainly due to the difficulty of validating tests when using ‘real-life’ specimens, in which only few, mostly intracellular bacteria (median, 0.5 CFU/mL blood) are present.^2^, ^14^ One method to increase the sensitivity of *S.* Typhi detection from blood is to use ox-bile as a selective culture media.^15^, ^16^ Ox-bile reduces both coagulation and serum complement killing activity and causes the selective lysis of human rather than *Salmonella* cells.^17^, ^18^ Recently, we developed a culture-PCR assay incorporating a brief pre-incubation in ox-bile along with PCR amplification of the *S.* Typhi flagellin gene, *fliC*.^19^, ^20^ Here, we have evaluated this culture-PCR assay as a diagnostic for detecting *S.* Typhi in the blood of healthy adult volunteers developing typhoid while participating in a human challenge model.^21^

## Methods

### Participants and challenge

Challenge of healthy adults with a single oral dose of a wild-type *S.* Typhi Quailes strain was performed in a dose-escalation study, as previously described.^4^, ^21^ Briefly, healthy consenting adult volunteers aged between 18 and 60 years were challenged by ingesting a fresh preparation of 10^3^ or 10^4^ CFU of *S.* Typhi suspended in sodium bicarbonate solution. After challenge, participants were reviewed daily for symptoms and signs of typhoid fever and clinical samples were collected (Table 1). If the study endpoint of typhoid diagnosis (TD) was reached, additional samples were collected and antimicrobial treatment was initiated. All remaining participants were given antimicrobial treatment on day 14. TD criteria were clinical (temperature ≥38 °C sustained for 12 h) and/or microbiological (positive blood culture).

### Diagnostic blood culture (reference standard)

Blood for culture was collected daily for 14 days or up to 96 h after TD, whichever was the later, and processed according to national standard methods as previously described.^21^, ^22^ At all time points after challenge 10 mL blood was collected, except at TD when this was reduced to 5 mL (Table 1). Stool culture was performed as previously described.^21^ Bacterial isolates were identified by phenotypic, biochemical and serological testing according the Kauffmann-White classification, following standard methods.^23^, ^24^

### Culture-PCR assay

To perform the culture-PCR assay, 5 mL of heparinised peripheral venous blood was collected at daily after challenge (Table 1), and performed as previously described.^19^, ^20^, ^25^ Briefly, blood was added to culture media (20 mL 3% (w/v) ox bile/tryptone soya broth containing 1.5 μL micrococcal nuclease) and incubated for 5 h (37 °C, 220 rpm New Brunswick, Excella e24). Bacteria were then concentrated by centrifugation at 6000 ×g for 20 min and the supernatant removed. DNA was extracted from the bacterial pellet using UltraClean™ BloodSpin™ kits following the manufacturer's instructions, except that elution of the final DNA was performed using 50 μL of pre-heated ‘Buffer 5’ (65 °C for 5 min) prior to centrifugation. PCR amplification was performed with primers targeting the *S.* Typhi *flagellin* gene, *fliC*-d (GenBank L21912,^26^) as described by Levy et al.^10^

Amplification reactions were performed in 50 μL volumes containing 10 μL DNA template and 0.2 μM each of H-for and Hd-rev primers (TopTaq PCR Master Mix Kit, Qiagen). Where required, distilled H_2_O and/or non-study DNA extracted from healthy donor blood was used as negative controls; genomic DNA extracted from *S.* Typhi Quailes strain culture was used as a positive control template. DNA amplification was performed using standard thermocycler equipment at: 95 °C for 5 min followed by 35 cycles of 93 °C for 30 s, 55 °C/30 s, and 72 °C/40 s and terminating with 1 cycle of 72 °C for 5 min. The specific target PCR amplicons could be observed at 763 bp when separated by 1% (w/v) ethidium bromide agarose gel electrophoresis and visualized using UV light transillumination. Amplicons were categorised as either being present (positive) or absent (negative) by visualisation with the naked eye.

### Blood inoculation experiments and detection limits

Prior to the investigation of clinical specimens, assay performance and laboratory sensitivity was evaluated using *S.* Typhi -negative whole blood (5 mL) containing known concentrations of *S.* Typhi DNA, added after the incubation step (Supplementary Fig. 1). The calculated sensitivity of PCR amplification was at least 0.015 ρg/μL, equivalent to a DNA starting concentration of 30 bacteria per reaction (each bacterium containing ∼5 fg DNA)^27^, ^28^ or ≥6 CFU/mL in the original starting blood volume, assuming no multiplication had taken place during incubation.

### Reporting and statistical analysis

Data pertaining to the diagnostic accuracy of the PCR assay in comparison with the predefined study endpoint of typhoid diagnosis (TD) or positive blood culture are reported according to the STARD criteria.^29^ The diagnostic accuracy of PCR or blood culture in comparison to TD are reported using sensitivity, specificity, positive and negative likelihood ratios, positive and negative predictive values and diagnostic odds ratio, each with a 95% confidence interval. The statistical significance of the differences in sensitivities of PCR and blood culture was assessed by discordant pairs analysis using a two-tailed binomial test. Data were analysed using Prism v6.0e (GraphPad Software Inc).

### Ethics

The challenge study was approved by the National Research Ethics Service (Oxfordshire Research Ethics Committee A, 10/H/0604/53) and was performed in accordance with the principles of the ICH-Good Clinical Practice guidelines and amendments. All study participants provided written informed consent in accordance with the Declaration of Helsinki. While all blood culture and PCR data were generated prospectively during the study, only blood culture results were used to make clinical management decisions.

## Results

### Performance of culture-PCR in a typhoid challenge study

Using a dose-escalation approach to determine the optimal challenge dose, 41 participants were challenged with either 10^3^ or 10^4^ CFU of *S.* Typhi Quailes strain, as previously described.^21^ Attack rates were 55% and 65% for each dose, respectively; the median day of illness onset was seven days after challenge.

**Figure 1:**
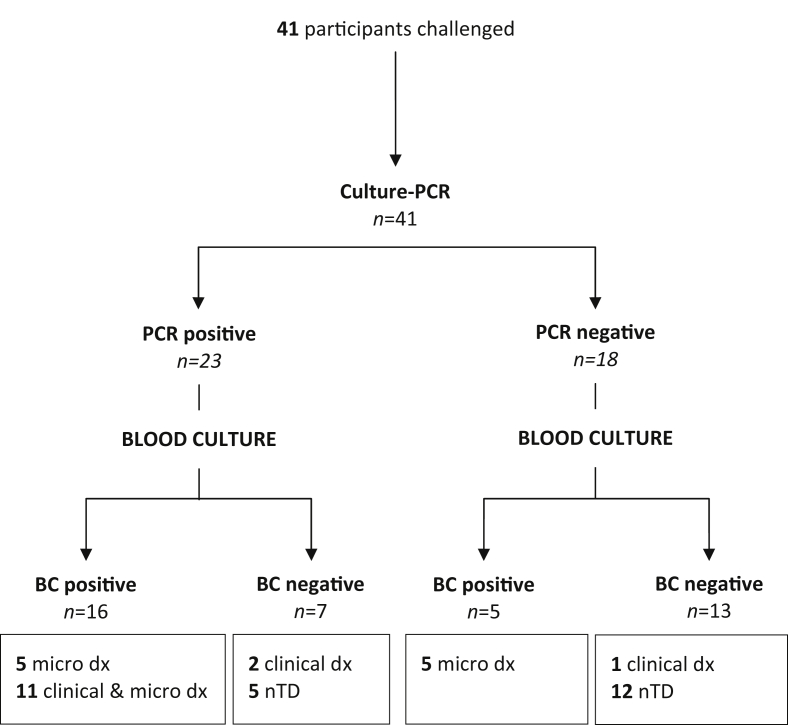
**STARD flowchart describing culture-PCR results in comparison with the reference standard (Blood culture, BC) for diagnosis of challenge study participants with typhoid infection after challenge.** nTD, non-typhoid diagnosed; clinical dx, clinical diagnosis; micro dx, microbiological diagnosis.

**Figure 2:**
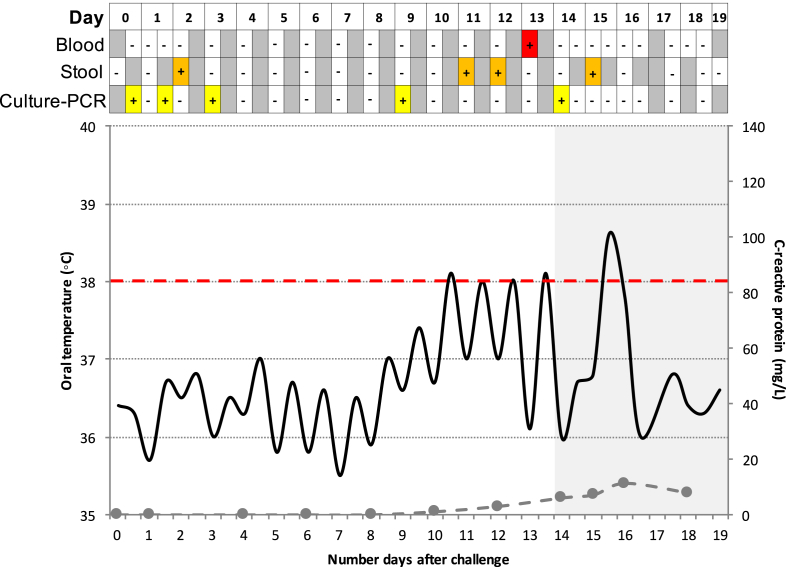
**Example of a challenge study participant who had several early positive culture-PCR results (*yellow squares*), in addition to early positive stool culture result (*orange squares*).** This participant was subsequently diagnosed with typhoid infection based on both microbiological and clinical criteria. *Red square*, positive blood culture; *grey squares*, no sample collected; *black line*, oral temperature; *dashed grey line*, C-reactive protein level; *shaded area*, antibiotic treatment initiated.

Overall, 684 serial samples were collected for culture-PCR from 41 challenge participants, 24 of whom were diagnosed with typhoid fever (TD). One individual was treated before day 14 based on symptoms alone without fulfilling the study TD criteria (included here in the nTD group, Supplementary Table 1); the remaining 16 participants were treated with antimicrobials at day 14 but did not develop infection, defined as not diagnosed with typhoid (nTD, Table 2). From the 684 samples collected 57 (8.3%) were positive by culture-PCR assay, and were collected from 23 study participants (Fig. 1). From three days after challenge onwards, nTD and TD participants yielded 11/203 (5.4%) and 37/332 (11.1%) positive culture-PCR assay results, respectively. After the initiation of antimicrobials 6/24 (25%) TD participants generated nine further positive culture-PCR assay results, of which 5/9 occurred within six hours of initiated antimicrobial treatment (e.g. Fig. 2).

**Figure 3:**
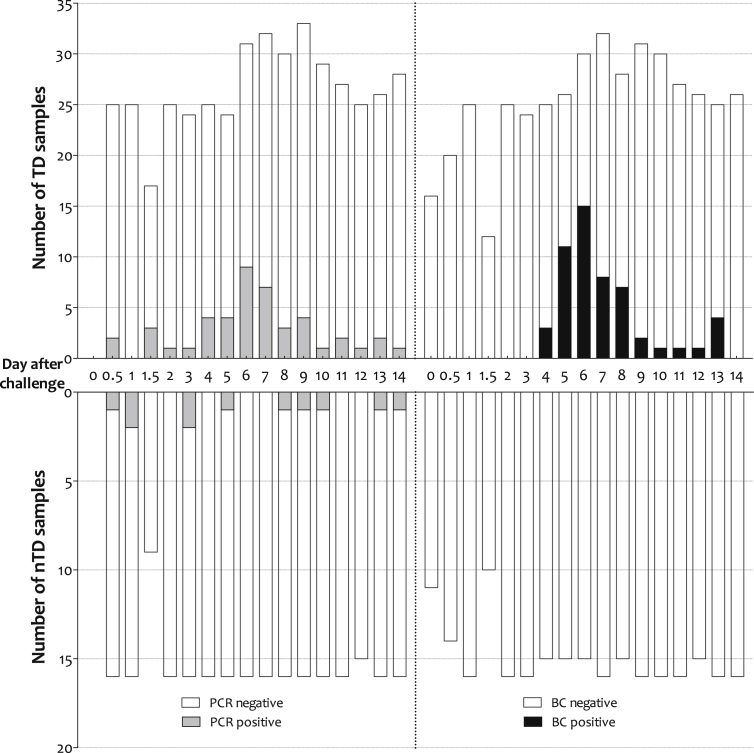
**Number of positive culture-PCR and blood culture samples collected after challenge by typhoid outcome.** The 6 and 12 h positive results have been pooled into the 0.5 day group. The maximum number of TD samples/day exceeds the number of TD participants as more than one sample was collected per day following initiation of antibiotic treatment. TD, typhoid diagnosed; nTD, non-typhoid diagnosed; PCR, culture-PCR assay; BC, blood culture.

In comparison with the culture-PCR methods, blood culture alone yielded 53/674 (7.9%) positive samples from the 41 study participants. Positive PCR amplifications were evident earlier in the challenge course than with culture, however the maximal day of blood culture and PCR positivity was six days after challenge for both assays (Fig. 3).

### Early primary DNAaemia in typhoid challenge participants

The earliest positive culture-PCR sample was collected only six hours after challenge; a further two participants were positive by 12 h. In total, nine positive samples from seven participants were obtained ≤48 h after the ingestion of *S.* Typhi with blood samples from a further two participants testing positive within 72 h (Table 2 and Fig. 3). There was no significant association between early stool shedding and culture-PCR assay positivity (*n* = 3/16, *p* = 0.63, Fisher's exact test; Fig. 2). Similarly, an early positive culture-PCR result was not predictive of subsequent development of typhoid infection (diagnostic odds ratio (DOR) 0.57, 95%CI 0.12–2.71, *p* = 0.69) or development of *S.* Typhi bacteraemia (DOR 0.50, 95%CI 0.10–2.44, *p* = 0.45).

### Confirmation of typhoid diagnosis

Of the 24 participants diagnosed with typhoid infection, 21/24 (87.5%) had a bacteraemia with or without reaching the clinical endpoint (Fig. 1, Supplementary Table 1). While culture-PCR and blood culture results concurred in participants with bacteraemia and fever, discrepancies arose in participants diagnosed by positive blood culture or temperature criteria alone. In three participants diagnosed by the clinical endpoint, 2/3 generated a positive culture-PCR result supporting the clinical diagnosis while all blood cultures remained negative throughout the challenge period (Fig. 1 and Supplementary Fig. 3). In addition, the participant treated early based on symptoms alone (i.e. no bacteraemia or fever) also had several positive culture-PCR assay results after the start of antimicrobial treatment.

In contrast, five participants had positive blood cultures on at least one occasion but remained PCR amplification negative throughout. In general, these five individuals had fewer positive blood cultures than those who generated positive blood cultures and were culture-PCR positive (mean, 2.00 vs. 2.73, respectively); this difference was non-significant (95%CI, −0.75 to 2.22, *p* = 0.31, T test).

Assuming that the predefined TD criteria were the best reference standard, culture-PCR demonstrated a sensitivity and specificity of 70 and 65%, respectively (Table 3). Despite forming part of the TD definition, the use of blood culture alone as a reference standard did not detect all cases of typhoid: routine blood culture was more sensitive and specific than culture-PCR; 87.5 and 100%, respectively. Interestingly, addition of the culture-PCR results to the study endpoint definitions suggested an overall attack rate after challenge of 73% rather than 59%. Discordant pairs analysis identified 12 mismatching culture-PCR and blood culture results, and confirmed that there was no significant difference in yield between culture-PCR and blood culture sensitivity if either was used alone to diagnose infection (*p* = 0.77, binomial test; Supplementary Table 2).

### Asymptomatic DNAaemia after typhoid challenge

In participants who remained nTD throughout the 14-day observation period, 6/17 generated ≥1 positive culture-PCR result (Fig. 1). Further investigation of participants' clinical features demonstrated that several had features indicative of the development of typhoid infection in this controlled challenge scenario (Supplementary Table 3). These features included a single elevated temperature reading within 12 h of the positive culture-PCR result (Supplementary Fig. 4), maximal symptom reporting on the day of the positive culture-PCR result (Supplementary Table 3) and the additional participant who was treated based on symptoms without meeting the TD endpoint definition. The remaining 2/6 participants had only mild symptoms, reporting constipation and a cough on the day of the positive result.

## Discussion

Direct detection of pathogen nucleic acid is a widely-used method to increase sensitivity, specificity and time-to-result in modern diagnostic laboratories.^30^, ^31^, ^32^ We have previously described the first culture-PCR assay for detecting *S.* Typhi in the modern era^20^; here, we demonstrate the performance of this method, specifically designed to sensitively detect the *S.* Typhi *fliC*-d gene in the blood of participants undergoing typhoid challenge. While the direct comparison with blood culture suggested that the sensitivity of the assay was lower for participants reaching the typhoid diagnosis study endpoints, overall, the culture-PCR provided useful additional information compared with blood culture alone. This additional approach enabled the detection and confirmation of typhoid infection cases that would have been missed by blood culture. In using a human typhoid challenge model to perform the evaluation, we have provided unique insights into the natural history of typhoid infection. These include evidence for the frequency of asymptomatic infection after exposure, and the confirmation of bacterial DNA circulation in blood soon after exposure.

The application of PCR-based laboratory methods to confirm clinical diagnoses of typhoid fever are not commonly reported, despite the appeal of detecting non-cultivable bacteria. This is of special relevance in most endemic settings, where antimicrobial pre-treatment or immune interference are common and likely reduce culture sensitivity still further. In this study we demonstrate a PCR sensitivity of 70% using selective pre-incubation in ox-bile of a 5 mL blood sample, compared with the study defined endpoints. As reported previously, the median quantitative bacterial loads at diagnosis are 0.47 and 1.10 CFU/mL in participants ingesting the 10^3^ or 10^4^ CFU dose, respectively.^21^ With our estimation of a lower detection limit of 6 CFU/mL, it is not surprising that the assay may have failed to detect some cases. The extreme challenge posed by detecting such a small number of bacteria has been recently demonstrated in a field study evaluating a sensitive qPCR, capable of detecting ∼1 CFU/mL, which, despite favourable performance in developmental stages demonstrated field performance of 40% sensitivity and 91% specificity.^33^

While the detection of bacterial DNA may be increased by pre-incubation of clinical material prior to performing DNA extraction,^27^, ^34^, ^35^ disadvantages to this method include loss of the ability to accurately quantify bacterial numbers in the blood directly, prolongation of assay time and the requirement for microbiological culture facilities. Nevertheless, selective culture to release viable bacteria from the blood intracellular compartment has been shown to produce an almost 2-fold rise in bacterial numbers.^36^ While broths containing 10% and more of ox-bile have been shown to be at best bacteriostatic for *S.* Typhi growth, our recent work identified that, at an optimal concentration of ∼2.4% and with 5-h incubation, a significant increase in bacterial numbers may be achieved (Supplementary Table 4).^18^, ^19^ The addition of micrococcal nuclease during the extraction process to remove remaining human DNA further improved assay sensitivity.^20^

Our identification of participants with asymptomatic infection, i.e. participants who had evidence of DNA in the blood (positive culture-PCR result) or bacterial shedding in stool (positive stool culture) in the absence of fever or clinical signs of infection, may represent false positive assay results. While various *S.* Typhi targets have been used previously, Song and colleagues first described the use of PCR to detect flagellin sequences in 1993.^12^ Flagellin expression is monophasic in several *Salmonella* sp. including *S.* Typhi, and the phase-1 antigen ‘d’ is found in many species. While the end regions of *fliC*-d (previously *H1*-d) are identical between species, there are two hypervariable regions (IV and VI) unique to *S.* Typhi and similar to *S.* Muenchen.^26^ Selecting primers targeting this region should therefore result in good specificity with little overlap with environmental or other *Salmonella* species.

Identification of probable asymptomatic, subclinical typhoid patients has been previously noted in other susceptible patient groups including the historical typhoid challenge studies performed in Maryland.^37^ A study in Cambodia, for example, identified a subpopulation among children <16 years old presenting with fever who were culture-negative but positive using a real-time PCR assay.^38^ Of note, this subpopulation was younger, had a shorter duration of illness prior to presentation and presented with fewer features characteristic of typhoid infection. ‘Incomplete immunity’ and earlier presentation with illness are common to both patient/participant groups which may reflect higher bacterial loads.

The occurrence of a primary, subclinical, bacteraemia that results in the dissemination of bacteria to the lymphoreticular system has long been speculated.^7^, ^16^ While early studies demonstrated bacteraemia in patients and challenge study participants as early as 4 or 3 days, respectively,^16^, ^37^ these cases likely represented early true infection. Individuals were febrile and went on to develop overt clinical infection, probably as a result of high exposure dose, which has been shown to correlate to shorter incubation periods.^21^ Similar results were observed in studies performed in chimpanzees.^39^ Our culture-PCR results suggest that *S.* Typhi DNA is detectable in blood within 48 h after ingestion; data that are supported by corresponding increases in plasma cytokines and host gene expression activity.^40^ Factors affecting the outcome of these early invasion events, which probably occur more frequently than was recognised here as demonstrated by the ubiquitous cytokine signature found in challenged participants, are uncertain but probably include the host inflammatory responses and early immune responses.

There are known limitations to reporting of detecting *S.* Typhi DNA from clinical samples, which include the inappropriate validation of the methodology, using nested primers and use of archived samples rather than a real time comparison between culture and PCR technique.^13^ While some of these may be overcome by using real-time PCR, these techniques have reiterated the difficulty of amplifying very low DNA copy numbers which suggests its continued inferiority to standard bacterial culture.^13^ Overreliance on culture methods as the true reference standard is known to be problematic,^33^ however, and therefore corroborating clinical and stool culture data is also valuable.^41^

While PCR is a relatively commonly performed technique in most diagnostic laboratories, including those in less well-resourced settings, the pre-culture step, which is vital to increase assay sensitivity, does not yet render the procedure beyond cross-infection (or cross-contamination) risk. The highly selective nature of ox-bile, while suitable for *S.* Typhi and other bile resistant organisms, means that this assay is of questionable applicability to most settings where clinical material is scant and *S.* Typhi is not the predominant pathogen.^6^ Alternative lysing agents have been proposed, including saponin (used for bacterial blood quantification in this study) and digitonin, which may produce cell lysis without loss of bacterial viability.^42^, ^43^ In the specific context of vaccine field or probe studies, however, such a ‘mono-directional’ assay, possibly with the additional of targeted *S.* Paratyphi A primers,^44^ may offer additional support to validate efficacy at least in small scale studies.

An important limitation of our assay was the volume of blood required to perform the test. While the lower volumes may be used, the stochastic nature of sampling such low-density bacteraemic blood will necessarily result in a reduction in sensitivity. It is also important to note the difference between blood volumes used in our evaluation: blood culture was performed with 10 mL whereas the culture-PCR assay was performed with 5 mL. While detection of bacteraemia was key to the endpoint for the clinical study, ethical and physiological limitations to sampling meant that a matched volume could not be collected at every time point. This likely underestimates the performance of our culture-PCR assay.

In conclusion, a selective culture-PCR assay targeting the flagellin gene, while less sensitive than optimally performed blood culture for the detection of participants developing typhoid infection after challenge, provides useful additive diagnostic information regarding the outcome of typhoid challenge. Performing evaluation of newly available diagnostics in the context of a human challenge study highlights important features of the natural history of typhoid infection. These include the frequency of asymptomatic cases and new evidence for primary bacteraemia occurring soon after ingestion.

## Funding

Funding for the challenge study was provided by a Wellcome Trust Strategic Translational Award (grant number 092661 to AJP; www.wellcome.ac.uk). Additional specific funding for this work was provided by Jesus College, Oxford (Graduate Scholarship to TCD).

TCD, CJ, CJB, CSW, AJP are supported by the NIHR Biomedical Research Centre (Oxford University Hospitals NHS Trust, Oxford; Clinical Research Fellowships to CSW and TCD); CJB is a Marie Curie Research Fellow supported by the European Union (FP7); AJP is a Jenner Investigator and James Martin Senior Fellow. SB is a Sir Henry Dale Fellow, jointly funded by the Wellcome Trust and the Royal Society (100087/Z/12/Z).

## Contributions

Conceived and designed the experiments: LZ, AJP, TCD. Performed the experiments: TCD, CJ, CJB, CSW. Analyzed the data: TCD, CJB. Contributed reagents/materials/analysis tools: TCD, LZ, CJ, CSW, SB. Wrote the paper: TCD, LZ, CJ, CJB, SB, AJP.

## Conflict of interest

All authors report no conflict of interest.

## Figures and Tables

**Table 1:** Assay schedule and associated blood volumes for laboratory diagnostic tests performed during the study. A) Assays performed in all participants, and B), assays performed in participants reaching clinical or microbiological TD endpoint. Challenge: oral ingestion of 10^3^ or 10^4^ CFU *S*. Typhi Quailes strain suspended in 30 mL/0.53 g NaHCO_3_(aq). Antimicrobials: first-line, ciprofloxacin 500 mg twice daily for 14 days.

A) All participants																			
Day	0	0	0	1	1	2	3	4	5	6	7	8	9	10	11	12	13	14	28
Hour	0	6	12	0	12	–	–	–	–	–	–	–	–	–	–	–	–	–	–
Procedure	Challenge																	Antimicrobials started	Antimicrobials completed
Blood culture	–	10	10	10	10	10	10	10	10	10	10	10	10	10	10	10	10	10	–
Culture-PCR	–	5	5	5	5	5	5	5	5	5	5	5	5	5	5	5	5	5	

B) Additional time points in typhoid-diagnosed participants								
Timepoint + hours	TD + 0	TD + 6	TD + 12	TD + 24	TD + 36	TD + 48	TD + 72	TD + 96
Procedure	Antimicrobials started							
Blood culture	5	10	10	10	10	10	10	10
Culture-PCR	5	5	5	5	5	5	5	5

**Table 2:** Number (%) of culture-PCR positive samples identified during the study according to challenge outcome and day/time of sample collection. Samples from participants diagnosed with typhoid are further described by day relative to typhoid diagnosis (and antibiotic initiation) in 48-h blocks. nTD, non-typhoid diagnosed; TD, typhoid diagnosed.

	Challenge outcome, *n/N* (%)		
	nTD (17 participants)	TD (24 participants)	ALL (41 participants)
**Time point after challenge**			
Day 0 to Day 3	4/62 (6.5)	5/87 (5.7)	9/149 (6.0)
Day 3 onwards	11/203 (5.4)	37/332 (11.1)	48/535 (9.0)
Total	15/265 (5.7)	42/411 (10.2)	57/684 (8.3)
**Time relative to TD**			
>−72 h	–	2/57 (3.5)	–
−72 to −24 h	–	18/44 (40.9)	–
−24 to +24 h	–	15/39 (38.4)	–
+24 to +72 h	–	0/83 (0)	–
>72 h	–	2/71 (2.8)	–

**Table 3:**
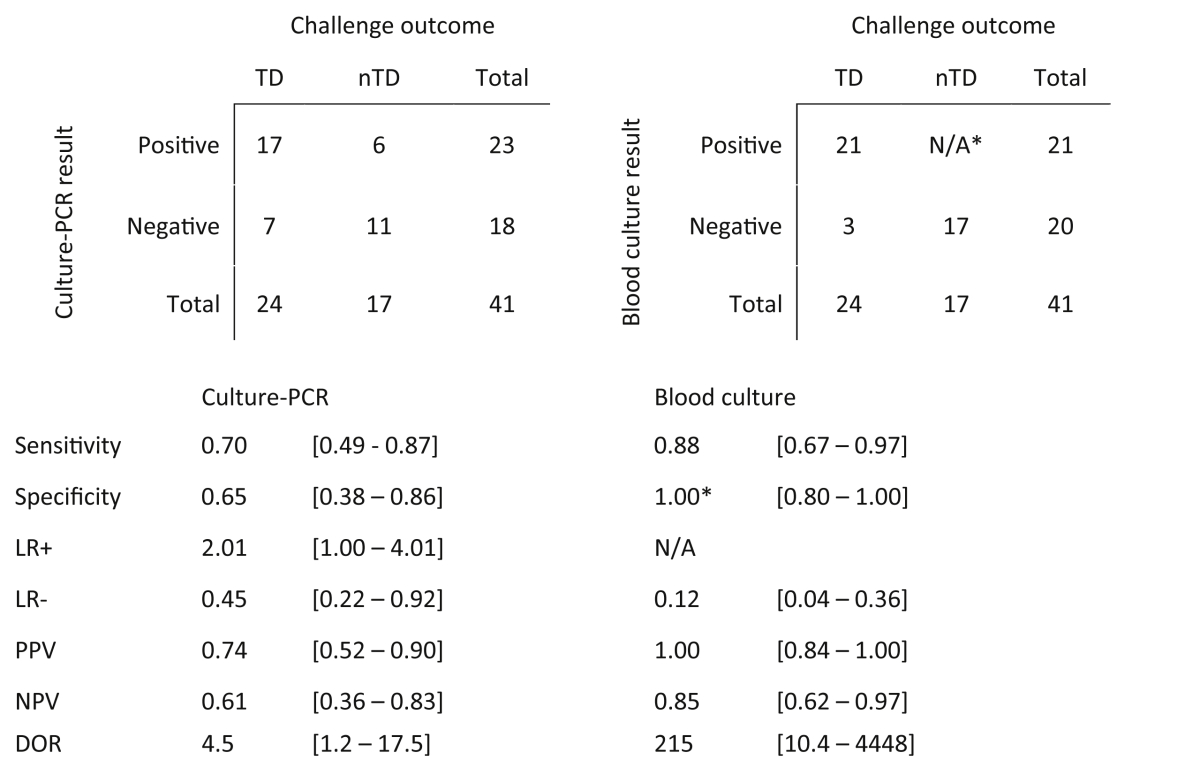
Contingency tables displaying estimates [95% CIs] of the sensitivity and specificity for culture-PCR and routine blood culture for diagnosing participants with typhoid infection during a challenge study. *Note that bacteraemia was one of the diagnostic criteria. TD, typhoid diagnosed; nTD, non-typhoid diagnosed; LR, likelihood ratio; PPV, positive predictive value; NPV, negative predictive value; DOR, diagnostic odds ratio.

## References

[1] Buckle G.C., Walker C.L., Black R.E. (2012). Typhoid fever and paratyphoid fever: systematic review to estimate global morbidity and mortality for 2010. J Glob Health.

[2] Baker S., Favorov M., Dougan G. (2010). Searching for the elusive typhoid diagnostic. BMC Infect Dis.

[3] Parry C.M., Hien T.T., Dougan G., White N.J., Farrar J.J. (2002). Typhoid fever. N Engl J Med.

[4] Darton T.C., Blohmke C.J., Pollard A.J. (2014). Typhoid epidemiology, diagnostics and the human challenge model. Curr Opin Gastroenterol.

[5] Parry C.M., Wijedoru L., Arjyal A., Baker S. (2011). The utility of diagnostic tests for enteric fever in endemic locations. Expert Rev Anti Infect Ther.

[6] Wain J., Hosoglu S. (2008). The laboratory diagnosis of enteric fever. J Infect Dev Ctries.

[7] World Health Organisation (2003). Background document: the diagnosis, treatment and prevention of typhoid fever. Department of immunization, vaccines and biologicals.

[8] Keddy K.H., Sooka A., Letsoalo M.E., Hoyland G., Chaignat C.L., Morrissey A.B. (2011). Sensitivity and specificity of typhoid fever rapid antibody tests for laboratory diagnosis at two sub-Saharan African sites. Bull World Health Organ.

[9] Ali A., Haque A., Haque A., Sarwar Y., Mohsin M., Bashir S. (2009). Multiplex PCR for differential diagnosis of emerging typhoidal pathogens directly from blood samples. Epidemiol Infect.

[10] Levy H., Diallo S., Tennant S.M., Livio S., Sow S.O., Tapia M. (2008). PCR method to identify *Salmonella enterica* serovars Typhi, Paratyphi A, and Paratyphi B among *Salmonella* isolates from the blood of patients with clinical enteric fever. J Clin Microbiol.

[11] Massi M.N., Shirakawa T., Gotoh A., Bishnu A., Hatta M., Kawabata M. (2003). Rapid diagnosis of typhoid fever by PCR assay using one pair of primers from flagellin gene of *Salmonella* typhi. J Infect Chemother.

[12] Song J.H., Cho H., Park M.Y., Na D.S., Moon H.B., Pai C.H. (1993). Detection of *Salmonella* Typhi in the blood of patients with typhoid fever by polymerase chain reaction. J Clin Microbiol.

[13] Nga T., Karkey A., Dongol S., Thuy H., Dunstan S., Holt K. (2010). The sensitivity of real-time PCR amplification targeting invasive *Salmonella* serovars in biological specimens. BMC Infect Dis.

[14] Wain J., Pham V.B., Ha V., Nguyen N.M., To S.D., Walsh A.L. (1998). Quantitation of bacteria in blood of typhoid fever patients and relationship between counts and clinical features, transmissibility, and antibiotic resistance. J Clin Microbiol.

[15] Coleman W. (1909). Short-duration typhoid fever. Am J Med Sci.

[16] Coleman W., Buxton B.H. (1909). The bacteriology of the blood in convalescence from typhoid fever. With a theory of the pathogenesis of the disease. J Med Res.

[17] Watson K.C. (1978). Laboratory and clinical investigation of recovery of *Salmonella* typhi from blood. J Clin Microbiol.

[18] Kaye D., Palmieri M., Rocha H. (1966 Mar). Effect of bile on the action of blood against *Salmonella*. J Bacteriol.

[19] Zhou L., Pollard A.J. (2010). A fast and highly sensitive blood culture PCR method for clinical detection of *Salmonella enterica* serovar Typhi. Ann Clin Microbiol Antimicrob.

[20] Zhou L., Pollard A.J. (2012). A novel method of selective removal of human DNA improves PCR sensitivity for detection of *Salmonella* Typhi in blood samples. BMC Infect Dis.

[21] Waddington C.S., Darton T.C., Jones C., Haworth K., Peters A., John T. (2014). An outpatient, ambulant-design, controlled human infection model using escalating doses of *Salmonella* Typhi challenge delivered in sodium bicarbonate solution. Clin Infect Dis.

[22] Health Protection Agency (2013).

[23] Health Protection Agency (2007). *Salmonella* identification: serotypes and antigenic formulae: Kauffmann-White Scheme 2007. London.

[24] Health Protection Agency (2011).

[25] Zhou L., Darton T., Waddington C.S., Pollard A.J., Annous B. (2012). Molecular diagnosis of enteric fever: progress and perspectives. Salmonella – distribution, adaptation, control measures and molecular technologies.

[26] Frankel G., Newton S.M., Schoolnik G.K., Stocker B.A. (1989). Unique sequences in region VI of the flagellin gene of *Salmonella* Typhi. Mol Microbiol.

[27] Zhu Q., Lim C.K., Chan Y.N. (1996). Detection of *Salmonella* Typhi by polymerase chain reaction. J Appl Bacteriol.

[28] Nair S., Poh C.L., Lim Y.S., Tay L., Goh K.T. (1994). Genome fingerprinting of *Salmonella* Typhi by pulsed-field gel electrophoresis for subtyping common phage types. Epidemiol Infect.

[29] Bossuyt P.M., Reitsma J.B., Bruns D.E., Gatsonis C.A., Glasziou P.P., Irwig L.M. (2003). The STARD statement for reporting studies of diagnostic accuracy: explanation and elaboration. Ann Intern Med.

[30] Darton T., Guiver M., Naylor S., Jack D.L., Kaczmarski E.B., Borrow R. (2009). Severity of meningococcal disease associated with genomic bacterial load. Clin Infect Dis.

[31] Gray J., Coupland L.J. (2014). The increasing application of multiplex nucleic acid detection tests to the diagnosis of syndromic infections. Epidemiol Infect.

[32] Maurin M. (2012). Real-time PCR as a diagnostic tool for bacterial diseases. Expert Rev Mol Diagn.

[33] Tennant S.M., Toema D., Qamar F., Iqbal N., Boyd M.A., Marshall J.M. (2015). Detection of typhoidal and paratyphoidal *Salmonella* in blood by real-time polymerase chain reaction. Clin Infect Dis.

[34] Chaudhry R., Laxmi B.V., Nisar N., Ray K., Kumar D. (1997). Standardisation of polymerase chain reaction for the detection of *Salmonella* typhi in typhoid fever. J Clin Pathol.

[35] Teh C.S., Chua K.H., Puthucheary S.D., Thong K.L. (2008). Further evaluation of a multiplex PCR for differentiation of *Salmonella* paratyphi A from other salmonellae. Jpn J Infect Dis.

[36] Wain J., Pham V.B., Ha V., Nguyen N.M., To S.D., Walsh A.L. (2001). Quantitation of bacteria in bone marrow from patients with typhoid fever: relationship between counts and clinical features. J Clin Microbiol.

[37] Woodard W.E. (1980). Summary of 1886 subjects in Volunteer challenges.

[38] Moore C.E., Pan-Ngum W., Wijedoru L.P., Sona S., Nga T.V., Duy P.T. (2014). Evaluation of the diagnostic accuracy of a typhoid IgM flow assay for the diagnosis of typhoid fever in Cambodian children using a Bayesian latent class model assuming an imperfect gold standard. Am J Trop Med Hyg.

[39] Edsall G., Gaines S., Landy M., Tigertt W.D., Sprinz H., Trapani R.J. (1960). Studies on infection and immunity in experimental typhoid fever. I. Typhoid fever in chimpanzees orally infected with *Salmonella* typhosa. J Exp Med.

[40] Blohmke C.J., Darton T.C., Jones C., Suarez N.M., Waddington C.S., Angus B. (2016). Interferon-driven alterations of the host's amino acid metabolism in the pathogenesis of typhoid fever. J Exp Med.

[41] Storey H.L., Huang Y., Crudder C., Golden A., de los Santos T., Hawkins K. (2015). A meta-analysis of typhoid diagnostic accuracy studies: a recommendation to adopt a standardized composite reference. PLoS One.

[42] Wain J., Diep T.S., Ho V.A., Walsh A.M., Nguyen T.T., Parry C.M. (2008). Specimens and culture media for the laboratory diagnosis of typhoid fever. J Infect Dev Ctries.

[43] Murray P.R., Spizzo A.W., Niles A.C. (1991). Clinical comparison of the recoveries of bloodstream pathogens in Septi-Chek brain heart infusion broth with saponin, Septi-Chek tryptic soy broth, and the isolator lysis-centrifugation system. J Clin Microbiol.

[44] Zhou L., Jones C., Gibani M.M., Dobinson H., Thomaides-Brears H., Shrestha S. (2016). Development and evaluation of a blood culture PCR assay for rapid detection of *Salmonella* paratyphi A in clinical samples. PLoS One.

