# Supplementary material for: Blood culture-PCR to optimise typhoid fever diagnosis after controlled human infection identifies frequent asymptomatic cases and evidence of primary bacteraemia
Source: J Infect. 2017 Apr;74(4):358–66. doi: 10.1016/j.jinf.2017.01.006 (PMC5345565; doi:10.1016/j.jinf.2017.01.006)
Supplement: Supplementary file 1 [file mmc1.docx]

|  | **Participant Identifier (Pt Id)** | | | | | | | | | | | | | | | | | | | | | | | | | | | | | | | | | | | | | | | | |
| --- | --- | --- | --- | --- | --- | --- | --- | --- | --- | --- | --- | --- | --- | --- | --- | --- | --- | --- | --- | --- | --- | --- | --- | --- | --- | --- | --- | --- | --- | --- | --- | --- | --- | --- | --- | --- | --- | --- | --- | --- | --- |
|  | 776 | 853 | 186 | 534 | 119 | 977 | 225 | 253 | 161 | 483 | 313 | 607 | 438 | 140 | 740 | 191 | 823 | 157 | 986* | 368 | 872 | 911 | 369 | 989 | 679 | 852 | 802 | 909 | 107 | 749 | 849 | 746 | 474 | 312 | 414 | 105 | 724 | 681 | 680 | 723 | 768 |
| **TD** |  |  | X |  |  |  |  |  |  |  |  |  |  |  |  |  | X |  |  | X | X | X | X | X | X | X | X | X | X | X | X | X | X | X | X | X | X | X | X | X | X |
| ***clinical*** |  |  | X |  |  |  |  |  |  |  |  |  |  |  |  |  | X |  |  | X |  | X |  |  |  |  | X |  | X | X |  |  | X | X | X | X | X |  | X | X |  |
| ***micro*** |  |  |  |  |  |  |  |  |  |  |  |  |  |  |  |  |  |  |  |  | X |  | X | X | X | X |  | X |  |  | X | X |  |  |  |  |  | X |  |  | X |
|  |  |  |  |  |  |  |  |  |  |  |  |  |  |  |  |  |  |  |  |  |  |  |  |  |  |  |  |  |  |  |  |  |  |  |  |  |  |  |  |  |  |
| **Blood cx.** |  |  |  |  |  |  |  |  |  |  |  |  |  |  |  |  |  |  |  |  | X | X | X | X | X | X | X | X | X | X | X | X | X | X | X | X | X | X | X | X | X |
| **Stool cx.** |  |  |  |  |  |  |  |  |  |  |  |  |  |  |  |  |  | X | X | X |  |  |  | X | X | X | X | X | X | X | X | X | X | X | X | X | X | X | X | X | X |
| **PCR** |  |  |  |  |  |  |  |  |  |  |  | X | X | X | X | X | X |  | X | X | X | X | X |  |  |  |  |  |  | X | X | X | X | X | X | X | X | X | X | X | X |

**Supplementary Tables and Figures**

**Supplementary Table 1. Summary of participant data describing challenge outcome, endpoint criteria reached and laboratory diagnostic tests.** The reason for reaching the typhoid diagnosis endpoint was identified by a study physician at TD, and based on clinical or microbiological (***micro***) criteria. Cx, culture; PCR, culture-PCR assay. Only positive results from 3 days after challenge are indicated. * study participant treated prior to day 14 not meeting study defined endpoints for typhoid diagnosis.

**Supplementary Table 2. Contingency table comparing participants blood culture and culture-PCR results.**

|  |  | **Blood culture** | | |  |
| --- | --- | --- | --- | --- | --- |
|  |  | Positive | Negative | Total |  |
| **Culture-PCR result** | Positive | 16 | 7 | 23 |  |
|  | Negative | 5 | 13 | 18 |  |
|  | Total | 26 | 15 | 41 |  |

**Supplementary Table 3. A table describing the microbiological and clinical features of five participants with positive culture-PCR results who remained non-typhoid diagnosed after challenge.** Pt id., participant identifier; Day, number of days after challenge that the positive sample was collected on; Blood/stool, blood and stool culture results from samples collected contemporaneously; -, negative result. *see **Supplementary Figure 4**.

| **Pt Id.** | **Day** | **Blood/stool** | **Oral temperature, °C** | **Symptoms recorded** |
| --- | --- | --- | --- | --- |
| **438*** | 10 | -/- | 36.0  (38.3 in evening) | Generally unwell, loss of appetite and muscle pain |
| **140** | 8 | -/- | 36.6 | Mild generally unwell, abdominal pain and diarrhoea (most symptomatic day) |
|  | 13 | -/- | 36.4  (35.2 previous evening) | None recorded |
| **740** | 3 | -/- | 35.7 | Mild constipation only |
| **607** | 3 | -/- | 36.7 | Mild headache, generally unwell and cough (most symptomatic day) |
|  | 5 | -/- | 36.5 | Mild cough only |
| **191** | 9 | -/- | 36.6 | Mild cough only |

**Supplementary Table 4.** Bacterial growth rate in 2.4% Ox-bile/TSB media and 20mLs blood. Mean of 3 experiments. Adapted from Zhou and colleagues. Ref.[25]

| **Incubation time (hours)** | **CFU** |
| --- | --- |
| 0 | 3 |
| 1 | 4 |
| 2 | 17 |
| 3 | 105 |
| 4 | 209 |
| 5 | 4461 |

**Supplementary Figure 1. Salmonella Typhi fliC-D amplicons (763bp) on a 1% agarose gel.** Lanes: M, DNA marker; P1, positive control with 15ρg/μL *S.* Typhi DNA; P2, 1.5ρg/μL; P3, 0.15ρg/μL; P4, 0.015ρg/μL; N, negative control (distilled H_2_O); NB, negative blood control; PB, positive blood (typhoid study clinical sample).

| M | P1 | P2 | P3 | P4 | N | NB | PB |
| --- | --- | --- | --- | --- | --- | --- | --- |


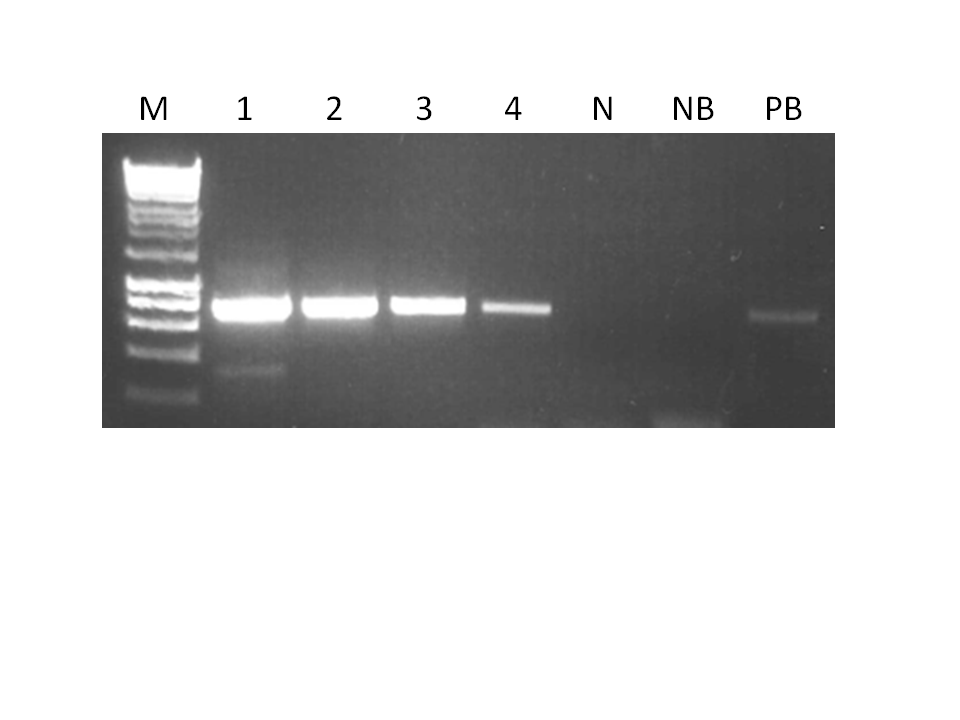


*fliC-*d

**Supplementary Figure 2. Representative Culture-PCR time course for an individual participant who was diagnosed with typhoid infection on Day 8 (TD).** A) Original PCR result – bands at TD+36, +48 and +72 hours were ambiguous, therefore the time course was repeated. B) Repeat PCR result using nested primers, which confirmed band seen at TD+36 hours.

| **A)** | | | | | | | | | | | | | |  | **B)** | | | | | | | | | | | | | |
| --- | --- | --- | --- | --- | --- | --- | --- | --- | --- | --- | --- | --- | --- | --- | --- | --- | --- | --- | --- | --- | --- | --- | --- | --- | --- | --- | --- | --- |
| Ladder | - control (dH_2_O) | + control 1 | + control 2 | + control 3 | - blood control | 6 hours | 12 hours | 24 hours | 36 hours | 48 hours | Day 3 | Day 4 | - *empty* - |  | Ladder | - control (dH_2_O) | + control 1 | + control 2 | + control 3 | - blood control | 6 hours | 12 hours | 24 hours | 36 hours | 48 hours | Day 3 | Day 4 | - *empty* - |


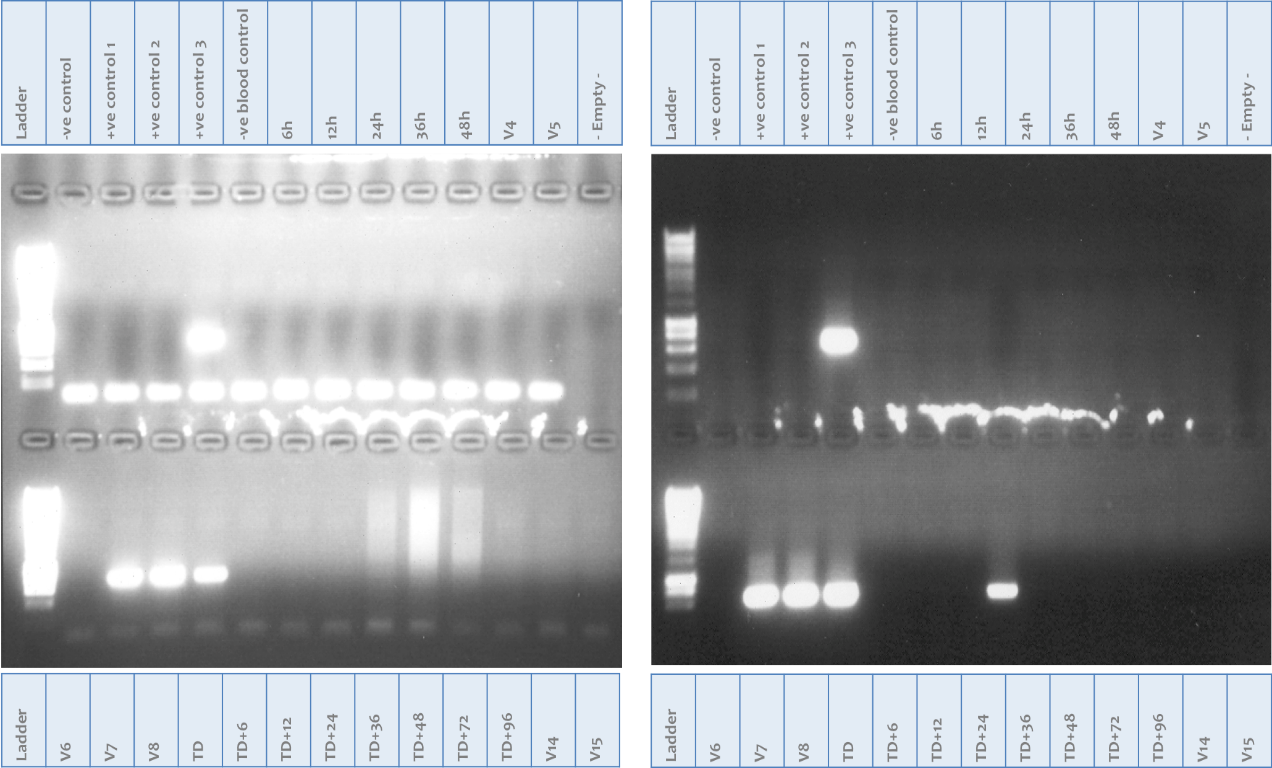


| Ladder | Day 5 | Day 6 | Day 7 | TD | TD +6 hours | TD +12 hours | TD +24 hours | TD +36 hours | TD +48 hours | TD +72 hours | TD + 96hours | Day 13 | Day 14 |  | Ladder | Day 5 | Day 6 | Day 7 | TD | TD +6 hours | TD +12 hours | TD +24 hours | TD +36 hours | TD +48 hours | TD +72 hours | TD + 96hours | Day 13 | Day 14 |
| --- | --- | --- | --- | --- | --- | --- | --- | --- | --- | --- | --- | --- | --- | --- | --- | --- | --- | --- | --- | --- | --- | --- | --- | --- | --- | --- | --- | --- |

**Supplementary Figure 3. Example of a challenge study participant who was diagnosed with typhoid infection based on clinical criteria (oral temperature ≥38^o^C for ≥12 hours) on Day 9 after challenge.** The only laboratory confirmation of the diagnosis was by culture-PCR (*yellow square*).

**Supplementary Figure 4**. Example of a participant (Pt Id. 438) who remained non-typhoid diagnosed but had a positive culture-PCR result (*yellow square*) on Day 10. This occurred on the maximal symptomatic day with an elevated recorded temperature (38.3°C, *black line*) that evening and a subsequent modest increase in CRP levels (*dashed grey line*).
